# The Oncogenic Role of KLF7 in Colon Adenocarcinoma and Therapeutic Perspectives

**DOI:** 10.1155/2023/5520926

**Published:** 2023-12-12

**Authors:** Zhenjia Li, Qi Liu

**Affiliations:** Department of Digestive Surgery, Shanghai Songjiang District Central Hospital, Shanghai 201600, China

## Abstract

Colon adenocarcinoma, a highly prevalent and aggressive form of colorectal cancer, necessitates a comprehensive understanding of its molecular mechanisms to identify potential therapeutic targets. The Krüppel-like factor 7 (KLF7), a transcription factor, has been associated with various malignancies, yet its specific role in colon adenocarcinoma remains largely unexplored. Here, we aimed to determine the expression and functional significance of KLF7 in colon adenocarcinoma. Our findings revealed a significant upregulation of KLF7 expression in colon adenocarcinoma tissues compared to adjacent normal tissues. Moreover, elevated KLF7 expression correlated with advanced tumor stage, lymph node metastasis, and poor overall survival in colon adenocarcinoma patients. Functional assays demonstrated that silencing KLF7 resulted in reduced cell proliferation, migration, and invasion, indicating its involvement in promoting tumor growth and metastasis. Additionally, we identified potential downstream targets of KLF7, including genes associated with cell cycle regulation and epithelial-mesenchymal transition. These results underscore the tumor-promoting role of KLF7 in colon adenocarcinoma, positioning it as a potential prognostic biomarker and therapeutic target for this aggressive disease.

## 1. Introduction

Colorectal cancer poses a substantial global health burden, with colon adenocarcinoma being the most prevalent subtype [1]. Despite advances in diagnostics and therapeutics, the molecular mechanisms driving the progression of colon adenocarcinoma remain incompletely elucidated. Transcription factors, pivotal in gene regulation and cellular processes, have been implicated in tumorigenesis [2]. The Krüppel-like factor 7 (KLF7), a member of the Krüppel-like factor family, has garnered attention for its involvement in various cancers, such as breast, lung, and pancreatic cancers [3–5]. However, the specific role of KLF7 in colon adenocarcinoma remains largely unexplored.

Recent evidence indicates that KLF7 functions as a transcriptional regulator and participates in multiple cellular processes, including cell proliferation, differentiation, and migration [6, 7]. Furthermore, several studies have implicated KLF7 in the regulation of critical pathways involved in cancer progression. KLF7 has been shown to modulate the expression of genes related to cell proliferation, cell death, epithelial-mesenchymal transition (EMT), and angiogenesis. These pathways significantly contribute to tumor growth, invasion, and metastasis. For example, KLF7 overexpression has been associated with increased cell proliferation in gastric cancer cells, implying an oncogenic role in promoting tumor growth [8]. On the other hand, studies in pancreatic cancer and lung have emphasized its function in tumor invasion and metastasis [3, 9]. Consequently, comprehending the expression patterns and functional implications of KLF7 in colon adenocarcinoma is vital for understanding its precise role and potential therapeutic implications.

In this study, our objective was to explore the expression and functional significance of KLF7 in colon adenocarcinoma. We hypothesized that KLF7 plays a tumor-promoting role in this aggressive malignancy. Gaining insight into the contribution of KLF7 to colon adenocarcinoma pathogenesis can provide valuable understanding of disease mechanisms and identify novel prognostic biomarkers and therapeutic targets. Our findings may pave the way for the development of targeted therapies to enhance the clinical management and outcomes of patients with colon adenocarcinoma.

## 2. Methods

### 2.1. Online Dataset

Gene expression data were obtained from The Cancer Genome Atlas (TCGA), a publicly accessible dataset. The dataset provided information on the expression levels of various genes, including the gene of interest, KLF7, across different stages. Utilizing the TCGA dataset allowed us to conduct a comprehensive investigation into the role of KLF7 in colon adenocarcinoma and explore its potential clinical implications.

### 2.2. Patient Cohort

We conducted a retrospective analysis using tissue samples from a cohort of patients diagnosed with colon adenocarcinoma at Shanghai Songjiang District Central Hospital. The study received approval from the Institutional Ethics Committee, and all patients provided written informed consent for the use of their tissue samples and clinical data for research purposes. We strictly adhered to patient confidentiality and data protection throughout the study.

### 2.3. RT-qPCR Analysis

To determine the RNA expression levels of KLF7 in the specimens from our medical center, we performed reverse transcription-quantitative polymerase chain reaction (RT-qPCR) analysis. Tumor tissues were collected and subjected to RNA extraction. The extracted RNA was then reverse transcribed into complementary DNA (cDNA). The resulting cDNA samples were subjected to qPCR using gene-specific primers for KLF7. The qPCR reactions were performed, and the amplification and detection of the target gene were monitored using a thermal cycler instrument. Data analysis was conducted to calculate the relative expression levels of KLF7, and statistical analysis was performed to assess differences in gene expression among different groups. Based on the median value, patients were divided into two groups: a low-KLF7 group (*n* = 87) and a high-KLF7 group (*n* = 88).

### 2.4. Cell Culture and Transfection

We cultured colon adenocarcinoma cell lines (HCT116 and SW480) in appropriate media supplemented with 10% fetal bovine serum (FBS) and antibiotics. To investigate the functional significance of KLF7, we transfected the cells with KLF7 shRNA or control shRNA according to the manufacturer's instructions. The efficiency of knockdown was confirmed through Western blot analysis.

### 2.5. In Vitro and In Vivo Functional Assays

Cell proliferation assay was performed to assess the impact of KLF7 knockdown on cellular behaviors using the CCK-8 method. Furthermore, an in vivo xenograft mouse model was utilized, by subcutaneously injecting tumor cells into nude mice, to evaluate the effect of KLF7 silencing on tumor growth. Tumor volume and weight were measured and recorded.

### 2.6. Statistical Analysis

Statistical analysis was carried out using SPSS software. Data are presented as mean ± standard deviation (SD). The significance of differences between groups was determined by Student's *t*-test or chi-square test. The Kaplan-Meier analysis and log-rank test were used to assess survival outcomes. Multivariate analysis was performed using Cox regression analysis to identify independent prognostic factors. A *P* value less than 0.05 was considered statistically significant.

### 2.7. Ethical Statement

The present study was conducted in accordance with the ethical guidelines and regulations of the Shanghai Songjiang District Central Hospital. All experimental protocols involving human subjects were approved by the Institutional Ethics Committee. Informed consent was obtained from each patient involved in the study for the use of their tissue samples and clinical data. Animal experiments were conducted following the guidelines set by the Institutional Animal Care and Use Committee to ensure the ethical treatment of animals.

## 3. Results

### 3.1. Expression and Clinical Significance of KLF7 in Online Datasets

**Figure 1:**
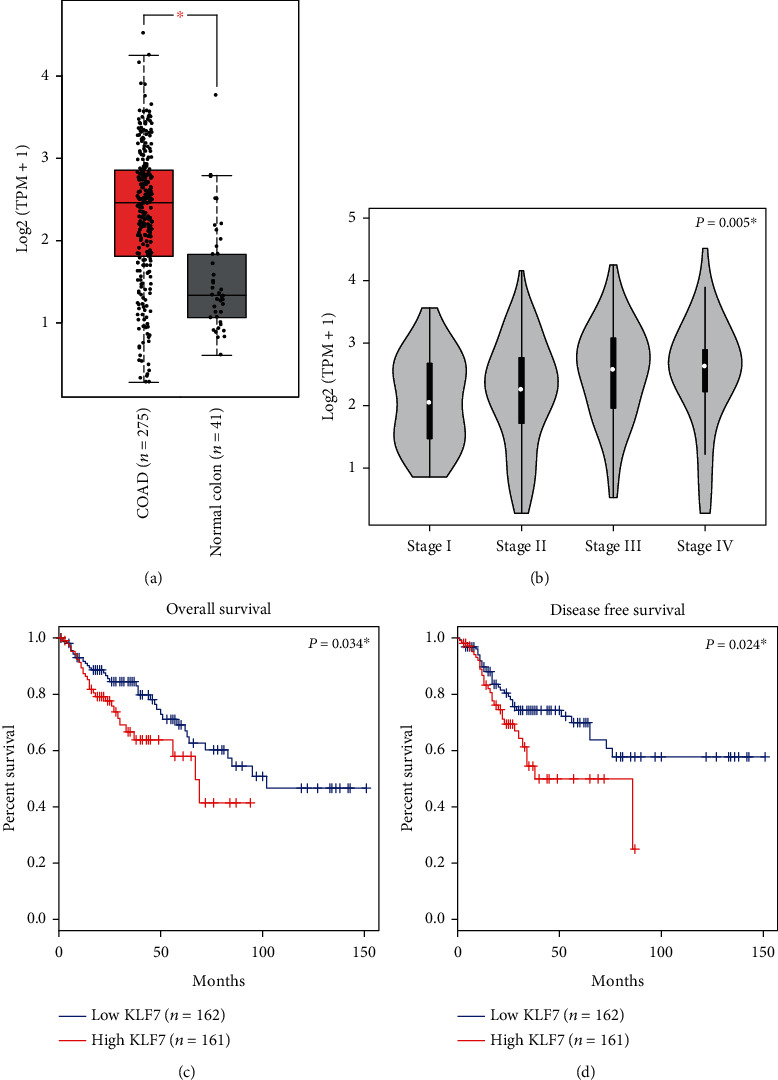
Expression and prognostic value of KLF7 in colon adenocarcinoma. (a) Comparison of KLF7 expression levels in colon adenocarcinoma (COAD) tissues and normal colon tissues in The Cancer Genome Atlas (TCGA) datasets, demonstrating the elevated expression of KLF7 in COAD. (b) Association between KLF7 expression and advanced stages of COAD. (c) Kaplan-Meier survival analysis showing the correlation between KLF7 expression levels and overall survival in COAD patients using data from the TCGA database. (d) Kaplan-Meier survival analysis illustrating the relationship between KLF7 expression levels and disease-free survival in COAD patients based on data from the TCGA database.

The mRNA expression level of KLF7 was extracted from the publicly available TCGA dataset, and its quantification was done as transcripts per kilobase million (TPM). A thorough analysis was performed to assess the expression patterns of KLF7 in colon adenocarcinoma (COAD) compared to nontumorous colon tissues. Using an unpaired Student's *t*-test, we observed a significant upregulation of KLF7 expression in COAD tissues compared to nontumorous colon tissues, as shown in Figure 1(a). Furthermore, we explored the association between KLF7 expression and tumor TNM stages, revealing a positive correlation between higher levels of KLF7 and more advanced tumor stages (Figure 1(b)). To gain further insights into the clinical significance of KLF7, the Kaplan-Meier survival analyses were performed using the TCGA dataset. These analyses demonstrated that increased KLF7 expression was linked to poorer overall survival (Figure 1(c)) and disease-free survival (Figure 1(d)) in COAD patients. These findings underscore the potential prognostic value of KLF7 and its involvement in the progression of colon adenocarcinoma.

### 3.2. Patient Characteristics

Table 1 presents the characteristics of the patients included in the study and their correlations with KLF7 expression in colon adenocarcinoma (COAD). A total of 175 COAD patients were enrolled, comprising 84 females and 91 males. The patients were categorized based on age, with 72 patients aged 65 or younger and 103 patients older than 65 years. Age range was 33-85 years at the time of diagnosis, with a median age of 68 years. Tumor size was divided into two groups: tumors smaller than 5.0 cm (*n* = 109) and tumors measuring 5.0 cm or larger (*n* = 66). Tumor size range was 0.1-15 cm with a median tumor size of 4.2 cm. In our analyses, we selected age 65 yrs and tumor size 5.0 cm as the cutoff values to distinguish the two groups because these two values are more popularly used in clinical practice. The location of tumors within the colon was classified as ascending colon (*n* = 53), transverse colon (*n* = 38), and descending-sigmoid colon (*n* = 84). The patient distribution among the four groups (differentiation grades III-IV and grades I-II) in terms of the number of patients in each group is as follows: 13 well differentiated: grade I; 131 moderately differentiated: grade II; 25 poorly differentiated: grade III; and 6 undifferentiated: grade IV. T stage, representing the extent of primary tumor invasion, was categorized as T1 (*n* = 23), T2 (*n* = 42), T3 (*n* = 86), and T4 (*n* = 24). The N stage, indicating lymph node involvement, was classified as N0 (*n* = 104), N1 (*n* = 52), and N2 (*n* = 19). Chemotherapy status was recorded, with 118 patients receiving no chemotherapy or being unsure and 57 patients receiving chemotherapy.

### 3.3. Correlation between KLF7 Expression and Clinicopathological Characteristics

The mRNA level of KLF7 in colon adenocarcinoma tissues was assessed using RT-qPCR. Based on the median value, 88 cases (50.3%) exhibited high levels of KLF7 expression, while 87 cases (49.7%) showed low expression. Subsequently, we compared differences regarding clinicopathological characteristics between the two groups. There was no significant difference in KLF7 expression based on sex (*P* = 0.404) or age (*P* = 0.711). However, noteworthy correlations were observed between KLF7 expression and specific clinicopathological parameters. Tumor size demonstrated a significant association with KLF7 levels (*P* < 0.001), with larger tumors displaying higher KLF7 expression. The relationship between KLF7 expression and tumor location did not reach statistical significance (*P* = 0.683), suggesting that tumor location within the colon may not influence KLF7 expression.

Regarding tumor differentiation grade, higher KLF7 expression was observed in poorly differentiated tumors compared to well-differentiated or moderately differentiated tumors (*P* = 0.032). Furthermore, KLF7 expression exhibited significant correlations with T stage (*P* < 0.001) and N stage (*P* < 0.001). Advanced T stages (T3 and T4) and lymph node involvement (N1 and N2) were associated with elevated levels of KLF7 expression. Additionally, the administration of chemotherapy was found to be associated with increased KLF7 expression (*P* = 0.001).

These findings indicate that KLF7 expression is linked to specific clinicopathological features in COAD patients. Elevated KLF7 expression is associated with larger tumor size, poor differentiation, advanced T and N stages, and receipt of chemotherapy. These correlations provide valuable insights into the potential role of KLF7 in COAD progression and its implications as a prognostic marker and therapeutic target.

### 3.4. Prognostic Significance of KLF7 Expression

**Figure 2:**
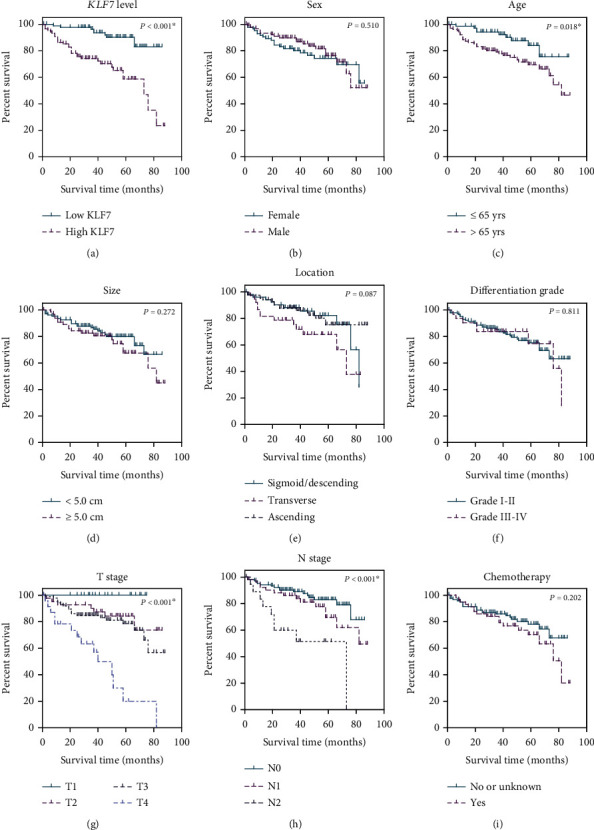
Prognostic value of KLF7 and clinicopathological parameters in COAD. Kaplan-Meier survival analysis of the COAD cohort, investigating the prognostic role of KLF7 expression level (a) and various clinicopathological parameters, including patients' sex (b), age (c), tumor size (d), tumor location (e), differentiation grade (f), T stage (g), N stage (h), and chemotherapy (i).

The prognostic significance of KLF7 expression in colon adenocarcinoma (COAD) was assessed using cancer-specific survival (CSS) analyses.  Table 2 and Figure 2 present the results of these analyses for various variables in the COAD cohort from your hospital. Regarding patient characteristics, no significant difference in 5-year CSS was observed based on sex (female: 73.4%; male: 76.4%; *P* = 0.510). However, age was found to be a significant factor, with patients aged ≤ 65 having a higher 5-year CSS (84.0%) compared to those aged > 65 (69.4%, *P* = 0.018).

Tumor-related factors also influenced survival outcomes. Tumor size showed a trend towards significance, with smaller tumors (<5.0 cm) associated with a higher 5-year CSS (79.9%) compared to larger tumors (≥5.0 cm, 67.5%, *P* = 0.272). Similarly, the location of the tumor within the colon exhibited a nonsignificant trend, with ascending colon tumors showing the highest 5-year CSS (82.3%, *P* = 0.087). Differentiation grade did not significantly impact survival, with grades I-II tumors demonstrating a 5-year CSS of 75.3% compared to grades III-IV tumors (74.6%, *P* = 0.811). However, the T stage was strongly associated with survival. T1 stage tumors showed a 100% 5-year CSS, whereas T4 stage tumors had the lowest 5-year CSS (19.9%, *P* < 0.001). Nodal involvement (N stage) was also a significant prognostic factor. Patients with no nodal involvement (N0) exhibited a higher 5-year CSS (82.8%) compared to those with N1 (69.5%) or N2 (51.3%) involvement (*P* < 0.001). Chemotherapy did not significantly impact survival outcomes, with patients who did not receive or had unknown chemotherapy showing a 5-year CSS of 78.0% compared to those who received chemotherapy (70.2%, *P* = 0.202).

Importantly, the level of KLF7 expression was found to have a significant impact on CSS. Patients with low KLF7 expression (*n* = 87) had a higher 5-year CSS rate of 90.2% compared to those with high KLF7 expression (*n* = 88) who had a CSS rate of 58.7% (*P* < 0.001).

Further multivariate analysis was conducted to examine the impact of various factors on CSS in COAD patients from your hospital (Table 3). In the Cox regression analysis, variables were selected for inclusion in the multivariate analysis based on a significance threshold of *P* < 0.05 in the univariate survival analysis. Variables meeting this criterion were included in the multivariate analysis, including age, T stage, N stage, and KLF7 level. The analysis revealed that the advanced T stage (HR = 1.747, 95% CI: 1.055-3.100, *P* = 0.047) was significantly associated with a higher risk of cancer-specific mortality. Additionally, patients with high KLF7 expression had a significantly elevated risk of cancer-specific mortality compared to those with low KLF7 expression (HR = 2.597, 95% CI: 1.070-6.301, *P* = 0.035). Age and N stage did not reach statistical significance in this analysis (both *P* > 0.05). These findings suggest that T stage and KLF7 expression level are independent prognostic factors for CSS in COAD patients. Considering these factors in patient risk stratification and treatment planning may be important.

### 3.5. Functional Role of KLF7 in Colon Adenocarcinoma Cells

**Figure 3:**
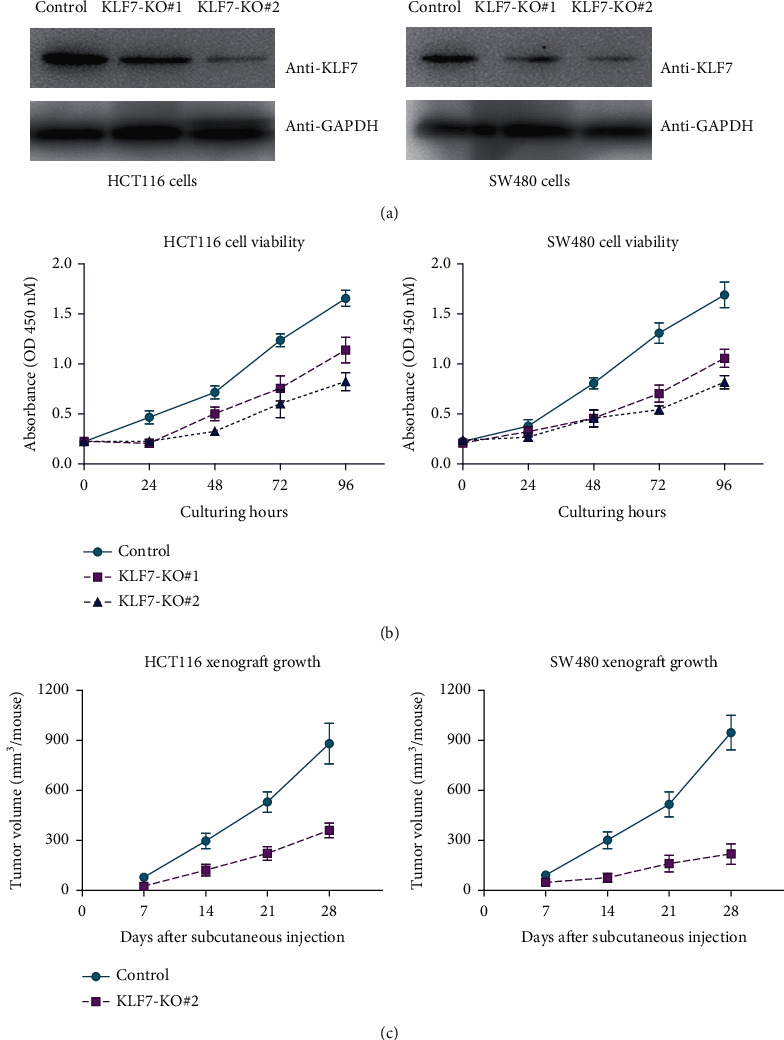
Functional effects of KLF7 knockdown in colon adenocarcinoma. (a) Western blot analysis confirmed the effective knockdown of KLF7 using specific shRNAs compared to the control scrambled shRNA in HCT116 and SW480 cell lines. (b) The CCK-8 assay demonstrated that silencing KLF7 resulted in a significant decrease in cell proliferation in colon adenocarcinoma cells. (c) Xenograft growth curves revealed a notable reduction in the growth rate of KLF7-knockout COAD cell-derived xenografts compared to control COAD cell-derived xenografts.

The functional role of KLF7 in colon adenocarcinoma was investigated both in vitro and in vivo. Effective knockdown of KLF7 was achieved using specific shRNAs, as confirmed by Western blot analysis in HCT116 and SW480 cell lines (Figure 3(a)). Silencing KLF7 led to a significant reduction in cell proliferation, as demonstrated by the CCK-8 assay conducted on colon adenocarcinoma cells (Figure 3(b)). Additionally, in vivo experiments using xenografts derived from KLF7-knockout COAD cells showed a notable decrease in tumor growth rate compared to the control COAD cells (Figure 3(c)).

**Figure 4:**
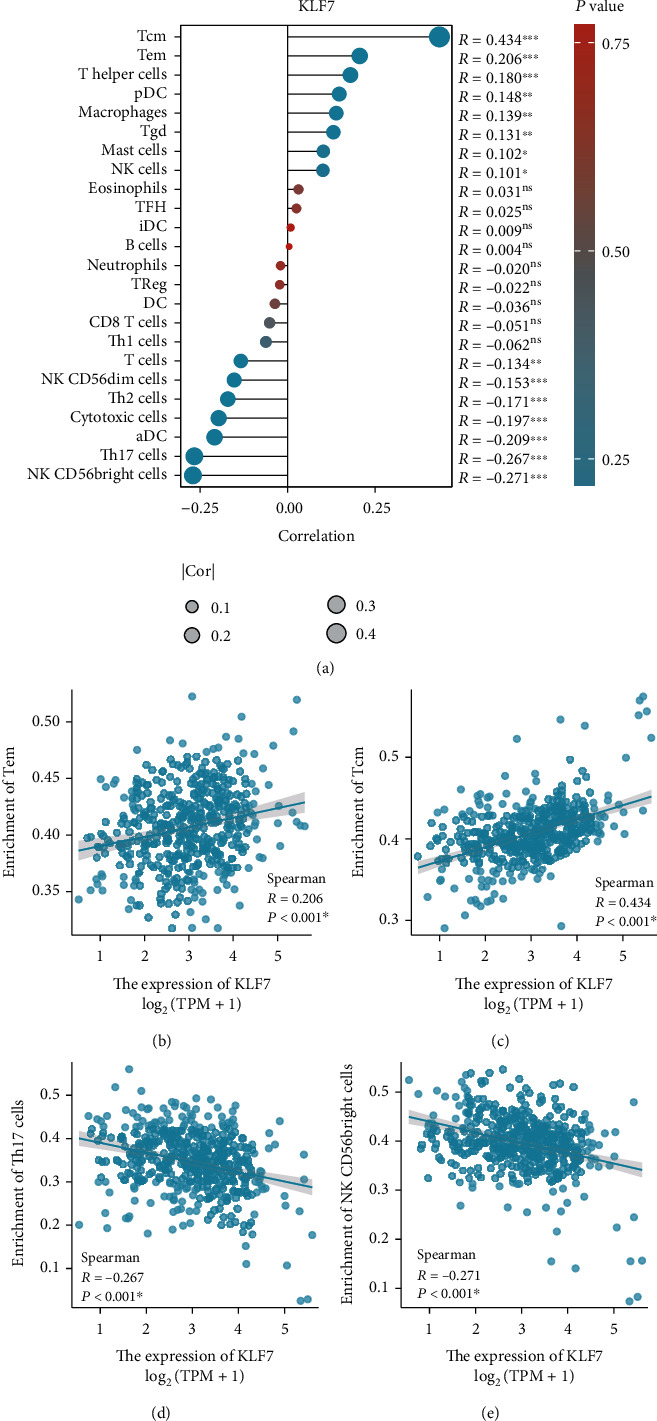
Correlation of KLF7 expression with immune cell infiltration in colon adenocarcinoma. (a) The analysis evaluated the expression level of KLF7 in relation to the infiltration of 24 distinct immune cell types in colon adenocarcinoma. (b–e) Representative correlations were observed between KLF7 expression and the infiltration of specific immune cell types, such as Tem cells, Tcm cells, Th17 cells, and NK CD56bright cells, indicating potential associations between KLF7 and immune responses in colon adenocarcinoma.

The relationship between KLF7 expression and immune cell infiltration in colon adenocarcinoma was also investigated. Analysis of 24 distinct immune cell types revealed potential associations between KLF7 expression and the infiltration of specific immune cells within the tumor microenvironment (Figure 4(a)). Correlations were observed between KLF7 expression and immune cell types such as Tem cells, Tcm cells, Th17 cells, and NK CD56bright cells (Figures 4(b)–4(e)), suggesting that KLF7 may play a role in modulating the immune response in colon adenocarcinoma. These findings imply that KLF7 may influence tumor progression and the response to immunotherapy by affecting the immune landscape of colon adenocarcinoma.

## 4. Discussions

Our study provides further evidence for the upregulation of KLF7 expression in colon adenocarcinoma tissues, consistent with its involvement in tumor progression and metastasis [10]. KLF7, as a member of the KLF family, has been implicated in various biological processes including cell proliferation, differentiation, and apoptosis. Elevated expression of KLF7 in colorectal cancer tissues comparing to adjacent normal tissues has also been reported in previous studies, indicating its relevance in promoting tumorigenesis [11, 12].

The correlation analysis conducted in our study reveals significant associations between high KLF7 expression and advanced tumor stage, lymph node metastasis, and poor tumor differentiation in colon adenocarcinoma. These findings suggest that KLF7 may contribute to the aggressive behavior of the cancer. Similar associations between KLF7 expression and clinicopathological features have been reported in other types of cancer, such as hepatocellular carcinoma, where high KLF7 expression has been linked to larger tumor size, lymph node metastasis, and advanced clinical stage [12]. This consistency across different cancer types supports the potential of KLF7 as a biomarker for assessing tumor aggressiveness and prognosis in colon adenocarcinoma.

Indeed, functional assays conducted in our study provided mechanistic insights into the tumor-promoting role of KLF7 in colon adenocarcinoma. Knockdown of KLF7 using shRNA resulted in a significant suppression of cell proliferation, indicating its involvement in promoting tumor growth. These findings are consistent with previous studies demonstrating the oncogenic functions of KLF7 in other malignancies, such as breast cancer and gastric cancer, where it has been shown to enhance cell proliferation, invasion, and angiogenesis [8].

The clinical data from our medical center and the TCGA dataset further support the significance of KLF7 expression in colon adenocarcinoma. High expression of KLF7 was associated with poorer survival outcomes in colon adenocarcinoma patients, and multivariate Cox regression analysis identified KLF7 expression as an independent prognostic factor for cancer-specific survival. Similar prognostic significance of KLF7 expression has been reported in other cancers, including oral squamous cell carcinoma and ovarian cancer. Incorporating KLF7 expression into existing prognostic models may improve their accuracy and enable personalized treatment strategies for colon adenocarcinoma patients.

The identification of KLF7 as a tumor-promoting factor in colon adenocarcinoma has important implications for both diagnosis and therapy. The upregulation of KLF7 expression in tumor tissues suggests its potential as a diagnostic biomarker for early detection and risk stratification. Immunohistochemical analysis of KLF7 expression in biopsy samples may aid in distinguishing malignant lesions from benign lesions or normal tissues. Moreover, the functional role of KLF7 in promoting cell proliferation, migration, and invasion suggests its therapeutic potential as a target for novel anticancer strategies. Future studies should focus on elucidating the molecular mechanisms underlying the tumorigenic effects of KLF7 in colon adenocarcinoma and investigating the efficacy of targeting KLF7 for therapeutic intervention.

While our study provides valuable insights into the role of KLF7 in colon adenocarcinoma, it is essential to acknowledge its limitations. Firstly, our analysis primarily relied on retrospective data, which may be subject to selection bias and confounding variables. Additionally, the study's sample size, although significant, could benefit from further expansion to enhance statistical power and generalize the findings to a broader population. Moreover, the functional assays performed in vitro and in vivo provide mechanistic insights but do not fully elucidate the complex molecular pathways through which KLF7 influences tumor progression. Further studies are needed to explore these mechanisms comprehensively. Finally, our investigation into the relationship between KLF7 expression and immune cell infiltration provides intriguing preliminary data but requires validation through more extensive immunological profiling.

## 5. Conclusions

Overall, our study adds to the existing body of knowledge regarding the role of KLF7 in cancer, particularly in colon adenocarcinoma. The upregulation of KLF7 expression and its associations with clinicopathological features highlight its potential as a diagnostic and prognostic marker. Further research is needed to elucidate the underlying mechanisms by which KLF7 contributes to tumor progression and metastasis and to explore its therapeutic implications in colon adenocarcinoma.

## Figures and Tables

**Table 1:** Correlations between *KLF7* level and COAD characteristics.

Variables	Case no.	*KLF7* level		*P* value
	(*n* = 175)	Low (*n* = 87)	High (*n* = 88)	
*Sex*				
Female	84	39	45	0.404
Male	91	48	43	
*Age (years old)*				
≤65	72	37	35	0.711
>65	103	50	53	
*Tumor size (cm)*				
<5.0	109	70	39	<0.001^∗^
≥5.0	66	17	49	
*Tumor location*				
Ascending colon	53	29	24	0.683
Transverse colon	38	18	20	
Descending-sigmoid colon	84	40	44	
*Differentiation grade*				
Grades I-II	144	77	67	0.032^∗^
Grades III-IV	31	10	21	
*T stage*				
T1	23	20	3	<0.001^∗^
T2	42	35	7	
T3	86	30	56	
T4	24	2	22	
*N stage*				
N0	104	69	35	<0.001^∗^
N1	52	15	37	
N2	19	3	16	
*Chemotherapy*				
No or unknown	118	69	49	0.001^∗^
Yes	57	18	39	

**Table 2:** Cancer-specific survival analyses of COAD cohort from our hospital.

Variables	Cases (*n* = 175)	5-year CSS (%)	*P* value
*Sex*			
Female	84	73.4%	0.510
Male	91	76.4%	
*Age (years old)*			
≤65	72	84.0%	0.018^∗^
>65	103	69.4%	
*Tumor size (cm)*			
<5.0	109	79.9%	0.272
≥5.0	66	67.5%	
*Tumor location*			
Ascending colon	53	82.3%	0.087
Transverse colon	38	67.9%	
Descending-sigmoid colon	84	75.2%	
*Differentiation grade*			
Grades I-II	144	75.3%	0.811
Grades III-IV	31	74.6%	
*T stage*			
T1	23	100%	<0.001^∗^
T2	42	84.2%	
T3	86	78.3%	
T4	24	19.9%	
*N stage*			
N0	104	82.8%	<0.001^∗^
N1	52	69.5%	
N2	19	51.3%	
*Chemotherapy*			
No or unknown	118	78.0%	0.202
Yes	57	70.2%	
*KLF7 level*			
Low	87	90.2%	<0.001^∗^
High	88	58.7%	

**Table 3:** Multivariate analysis for cancer-specific survival of COADs from our hospital.

Variables	Hazard ratio	95% confidence interval	*P* value
Age	1.935	0.876-4.276	0.103
T stage	1.747	1.055-3.100	0.047^∗^
N stage	1.316	0.807-2.144	0.271
*KLF7* level	2.597	1.070-6.301	0.035^∗^

## Data Availability

Data will be available upon reasonable request.
